# The DEAD-Box Protein Rok1 Coordinates Ribosomal RNA Processing in Association with Rrp5 in *Drosophila*

**DOI:** 10.3390/ijms23105685

**Published:** 2022-05-19

**Authors:** Jie Chen, Yuantai Huang, Kang Zhang

**Affiliations:** 1 Guangdong Provincial Key Laboratory of High Technology for Plant Protection, Plant Protection Research Institute, Guangdong Academy of Agricultural Sciences, Guangzhou 510640, China; 2 State Key Laboratory of Biocontrol, School of Life Sciences, Sun Yat-sen University, Guangzhou 510275, China; 18210700050@fudan.edu.cn

**Keywords:** RBPs, Rok1, Rrp5, pre-rRNA, ribosomal RNA

## Abstract

Ribosome biogenesis and processing involve the coordinated action of many components. The DEAD-box RNA helicase (Rok1) is essential for cell viability, and the depletion of Rok1 inhibits pre-rRNA processing. Previous research on Rok1 and its cofactor Rrp5 has been performed primarily in yeast. Few functional studies have been performed in complex multicellular eukaryotes. Here, we used a combination of genetics and developmental experiments to show that Rok1 and Rrp5, which localize to the nucleolus, play key roles in the pre-rRNA processing and ribosome assembly in *D. melanogaster*. The accumulation of pre-rRNAs caused by Rok1 depletion can result in developmental defects. The loss of Rok1 enlarged the nucleolus and led to stalled ribosome assembly and pre-rRNA processing in the nucleolus, thereby blocking rRNA maturation and exacerbating the inhibition of mitosis in the brain. We also discovered that *rrp5^4-2/4-2^* displayed significantly increased ITS1 signaling by fluorescence in situ hybridization, and a reduction in ITS2. Rrp5 signal was highly enriched in the core of the nucleolus in the *rok1^167/167^* mutant, suggesting that Rok1 is required for the accurate cellular localization of Rrp5 in the nucleolus. We have thus uncovered functions of Rok1 that reveal important implications for ribosome processing in eukaryotes.

## 1. Introduction

Ribosomes are ubiquitously found within organisms, and their primary role within cells is the catalysis of protein synthesis [1]. Proteins are essential in all organisms, as they carry out most of the cell’s essential functions. Therefore, ribosomal function is essential for life. Ribosome-catalyzed protein synthesis requires large amounts of energy and involves very complex processes [1,2,3,4]. Ribosomal RNA synthesis and processing are accompanied by ribosomal protein assembly. Ribosomal protein assembly involves more than 200 proteins. Most of the non-ribosomal proteins involved in this process belong to a variety of energy-consuming enzyme families [3], including the ATPase, RNA helicase, GTPase, kinase, and nuclease families [5]. While pre-rRNA is being transcribed, a variety of assembly factors (AFs), ribosomal proteins, and small nucleolar RNAs (snoRNAs) enter the nucleolus to participate in pre-rRNA folding, processing, and modification [6]. During the biogenesis of eukaryotic ribosomes, AFs and ribosomal proteins assemble with pre-rRNA to form preribosomal particles. After further maturation steps, the particles are transported from the nucleolus into the cytoplasm, where mature 60S and 40S subunits assemble into the 80S ribosomes and subsequently synthesize proteins [5,7].

In studies in yeast, researchers found that Rrp5, a special assembly factor, is involved both in the assembly of the ribosomal large subunit precursor (pre-60S) and the small subunit precursor (pre-40S) [8,9]. During rRNA processing, Rrp5 binds to pre-40S subunits and recruits other assembly factors to pre-40S subunits, including the DEAD box protein Rok1 [10,11]. After the function of Rrp5 in 40S subunit assembly is complete, it remains bound to the pre-60S subunit for its assembly [12,13].

Rrp5 is known to bind specifically to rRNA with the help of DEAD box protein Rok1 [14]. DEAD box proteins are a large class of RNA-dependent ATPases, which participate in various functions including gene expression regulation and RNA metabolism [15]. They carry out functions including unwinding RNA structures, annealing double-stranded nucleotide sequences, binding RNA, and remodeling RNA protein complex structures [16,17,18,19]. Although Rok1 is classified as an RNA helicase, in vitro experiments show that Rok1 does not have obvious RNA helicase activity [14,20]. It does show significant activity in regulating RNA double strand annealing [14]. Sohail et al. discovered the interaction between Rrp5 and Rok1 in rRNA processing in yeast [21]. Experiments in vivo and in vitro have verified that the Rrp5 protein forms a complex by contact with multiple structural regions of the Rok1 protein, found on the domains required for 40S subunit binding. Rok1 protein separates Rrp5 from the pre-40S subunit precursor by hydrolysis of its bound ATP, and transfers Rrp5 to the pre-60S subunit precursor, thus participating in the maturation process of the large subunit precursor [21].

Previous research on Rok1 and Rrp5 has focused mainly on yeast, and few functional studies have been performed using complex multicellular eukaryotes. The loss of Rok1 or Rrp5 leads to cell death in yeast [21], which is a single-cell organism, lacking in developmental processes. Rok1- and Rrp5-dependent effects on survival and development in complex organisms remain to be explored. Here, we use a combination of genetics and developmental experiments to show that Rok1 and Rrp5 exert key functions in the development of *D. melanogaster*. The accumulation of pre-rRNAs caused by *Rok1* depletion leads to the blocking of rRNA maturation and subsequently results in developmental defects. In addition, our results suggest that the loss of *Rok1* changes the cellular localization of Rrp5 in the nucleolus of multicellular eukaryotes. We have thus revealed functions of Rok1 that have important implications for ribosome assembly in multicellular organisms, as well as in yeast.

## 2. Results

### 2.1. Loss of Rok1 and Rrp5 Enlarges the Nucleolus in D. melanogaster

**Figure 1:**
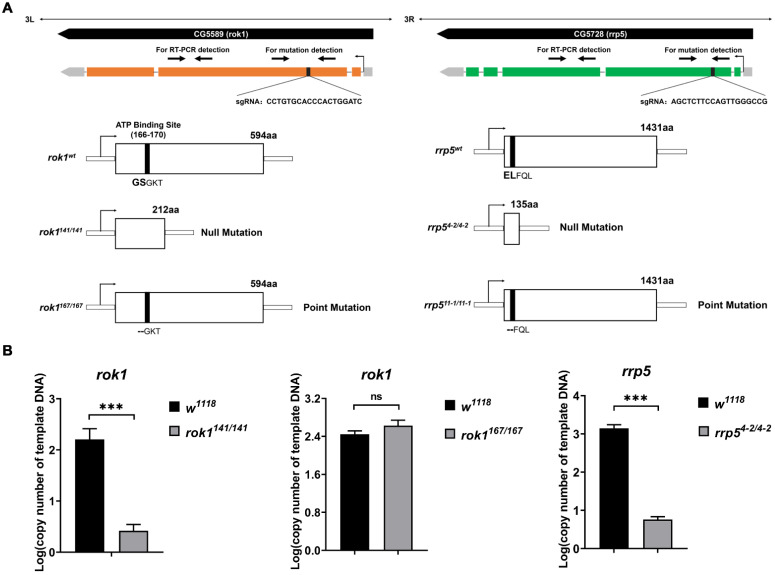
Mutation alleles of *rok1* (*rok1^141/141^* and *rok1^167/167^*) and *rrp5* (*rrp5^4-2/4-2^* and *rrp5^11-1/11-1^*) were generated by Crispr/Cas9. (**A**) Molecular nature of the mutations generated by Crispr/Cas9. Two null mutation alleles of *rok1* and *rrp5* (*rok1^141/141^* and *rrp5^4-2/4-2^*) were generated with the early termination of translation, and the point mutation allele of *rok1^167/167^* lost amino acids in the ATP-binding site, while *rrp5^11-1/11-1^* lost them in a non-functional domain. (**B**) The expression levels of *rok1* and *rrp5* were measured in *w^1118^*, *rok1^141/141^*, *rok1^167/167^*, and *rrp5^4-2/4-2^*. Thirty second-instar larvae of *w^1118^, rok1^141/141^*, *rok1^167/167^*, and *rrp5^4-2/4-2^* were pooled as one sample. The initial copy numbers of *rok1* and *rrp5* were calculated from the CT value of each cDNA sample after adjusting by the copy number of *Act5C*. *** indicate significant differences between the two groups at the *p* < 0.001 level (unpaired *t*-test, SPSS). Each point represents the mean ± SD (*n* = 3).

**Figure 2:**
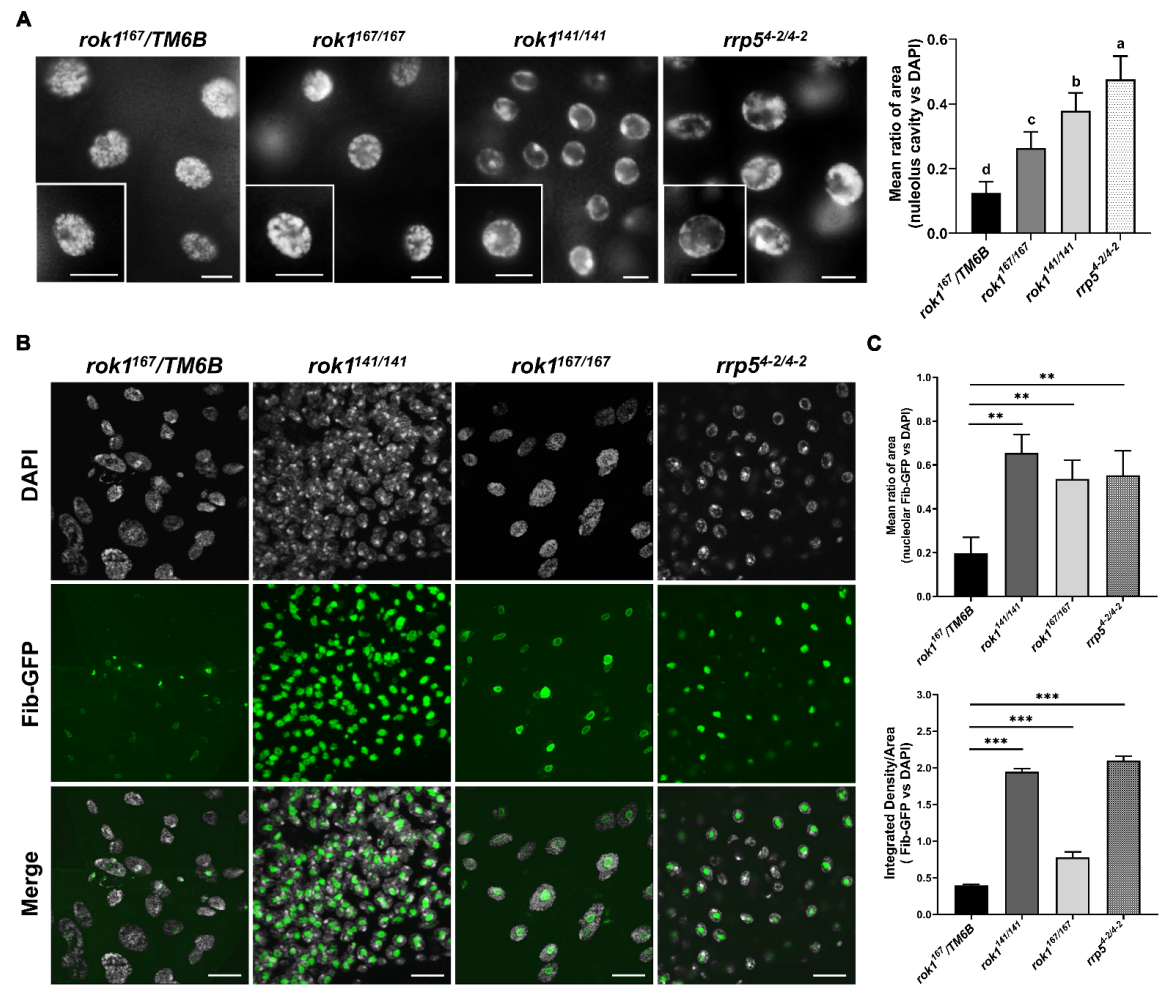
Loss of Rok1 and Rrp5 enlarges the nucleolus in *D. melanogaster*. (**A**) Nuclei from salivary glands in *rok1* and *rrp5* mutants. Salivary glands from second-instar larvae of *rok1^141/141^* and *rrp5^4-2/4-2^*, and third-instar larvae of *rok1^167^/TM6B* and *rok1^167/167^* were dissected, respectively. The nuclear genome is DAPI-stained. Scale bar represents 40 μm. Amplified areas marked with solid rectangles are shown as inserts. Mean ratio of area from salivary gland cells for each genotype is provided in panels to the right. Each point represents the mean ± SD (*n* = 35). Different letters indicate significant differences between areas in the various groups (*p* < 0.05, LSR, SPSS). (**B**) Localization of Fib, a nucleolar marker, in salivary gland of *rok1* and *rrp5* mutants. Salivary glands from second-instar larvae of *rok1^141/141^* and *rrp5^4-2/4-2^*, and third-instar larvae of *rok1^167^/TM6B* and *rok1^167/167^* were dissected, respectively. Scale bar represents 40 μm. (**C**) Mean ratio of area and fluorescence density of Fib-GFP in each genotype. Each point represents the mean ± SD (*n* = 35, for area; *n* = 9, for density). ** and *** indicate significant differences between the two groups at the *p* < 0.01 and *p* < 0.001 levels, respectively (unpaired *t*-test, SPSS).

Mutant alleles of *rok1 (rok1^141/141^* and *rok1^167/167^*) and *rrp5* (*rrp5^4-2/4-2^* and *rrp5^11-1/11-1^*) were generated by Crispr/Cas9. The molecular lesions in these four alleles are shown (Figure 1A). Two null mutation alleles of *rok1* and *rrp5* (*rok1^141/141^* and *rrp5^4-2/4-2^*) were generated with the early termination of translation, and the point mutation allele of *rok1^1^^67/^^167^* lost amino acids in the ATP-binding site, while *rrp5^11-1/11-1^* lost them in a non-functional domain. When we used qRT-PCR to measure the expression level of rok1 and rrp5, we observed significantly reduced expression in *rok1^141/141^* and *rrp5^4-2/4-2^* mutants (Figure 1B), as expected, given that *Rok1* and *Rrp5* are knockouts in null mutants. We noticed that the altered amino acid encoding sites in *rok1^167/167^* are in the ATP-binding pocket, which is conserved in yeast. We then produced *rok1* and *rrp5* double mutants using mutant alleles that we had generated. As shown in Table 1, death was expected in homozygous mutants except for *rrp5^11-1/11-1^*, but the stage of lethality was different for each mutant (Table 1). Interestingly, most *rok1^167/167^* individuals are also lethal during the pupal stage, although the *rok1* expression level was not decreased (Figure 1B). To assess larval development in *rok1* and *rrp5* mutants, we stained the salivary glands with DAPI and discovered that there were obvious “chromatin-poor” regions in the middle of the nucleus (Figure 2A). Compared with *rok1^167^/TM6B*, these areas were significantly larger in the three mutants (Figure 2A), leading to speculation that the nucleolus was enlarged. Finally, Fibrillarin (Fib), a nucleolar marker, was used to confirm that this region was the nucleolus. We observed that the GFP signals were also stronger in *rok1* and *rrp5* mutants by live fluorescent imaging of salivary glands expressing Fib-GFP (Figure 2B,C).

### 2.2. Rok1 Is Enriched in the Nucleolus and Responsible for rRNA Maturation

**Figure 3:**
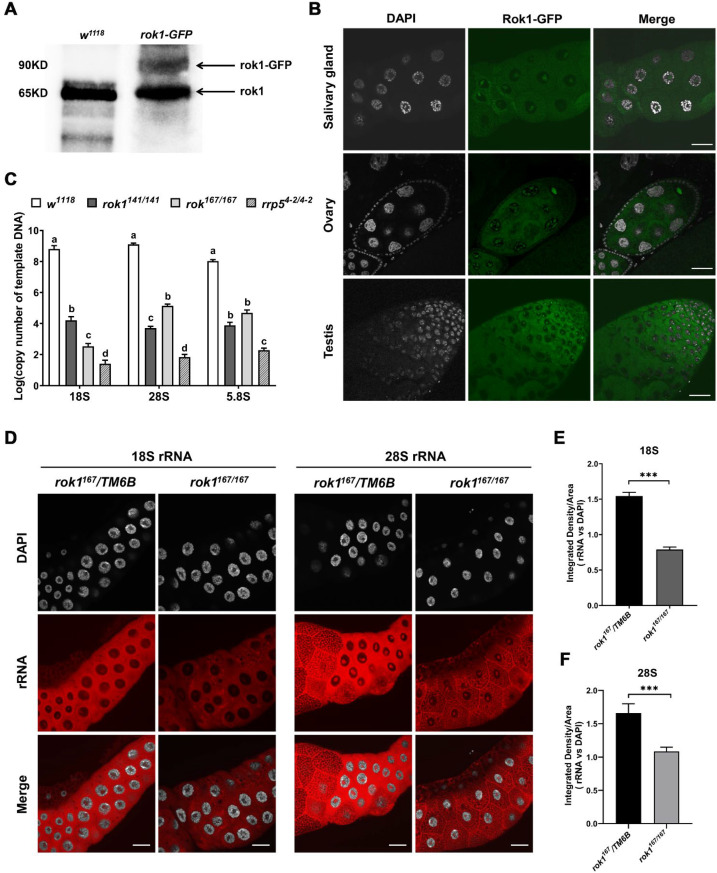
Rok1 is enriched in the nucleolus and is responsible for rRNA maturation. (**A**) Rok1 and Rok1-GFP levels were assayed by Western blotting. Total protein extracts (10 ug) from the embryos of *w^1118^* and *rok1-GFP* were loaded onto 10% SDS-PAGE gels. Arrows indicate the corresponding bands. (**B**) Overview of Rok1-GFP localization in the salivary gland, ovary, and testis. Salivary glands from third-instar larvae of *w^1118^*, ovaries, and testes from adult flies were dissected. Prominent Rok1 enrichment can be seen in a “chromatin-poor” region. Scale bar represents 40 μm. (**C**) The expression levels of mature rRNAs in *rok1* and *rrp5* mutants. Thirty second-instar larvae of *w^1118^, rok1^141/141^*, *rok1^167/167^*, and *rrp5^4-2/4-2^* were pooled as one sample. The initial copy numbers of 18S, 28S, and 5.8S were calculated from the CT value of each cDNA sample after adjusting by the copy number of *Act5C*. Each point represents the mean ± SD (*n* = 3). Statistics of results from 18S, 28S, and 5.8S were analyzed individually. Different letters indicate significant differences between levels in various groups (*p* < 0.05, LSR, SPSS). (**D**) FISH analyses of 18S rRNA and 28S rRNA localization in salivary glands. Salivary glands were dissected from the third-instar larvae of *rok1^167^/TM6B* and *rok1^167/167^*. A weaker rRNA signal is shown in the nucleus, and stronger signal is in the cytoplasm (DNA is in white and rRNA in red). Scale bar indicates 40μm. (**E,F**) Mean ratios of fluorescence density of 18S rRNA (**E**) and 28S rRNA (**F**). Each point represents the mean ± SD (*n* = 9). *** indicate significant differences between the two groups at the *p* < 0.001 (unpaired *t*-test, SPSS).

Rok1 is consistently enriched in the nucleoli of yeast cells [22]. We determined whether Rok1 is similarly distributed in the nucleoli of multicellular eukaryotes. To assess the Rok1 protein location within the cell, a Rok1-GFP fusion protein was constructed. As shown in Figure 3A, we detected distinct protein bands representing Rok1 and Rok1-GFP in the embryos *rok1-GFP* (Figure 3A). We then found that Rok1-GFP is lowly expressed in the tissues, although weak GFP signals in the salivary glands, ovaries, and testes of *w^1118^* flies were detected. It is worth noting that the prominent enrichment of Rok1 was observed in a “chromatin-poor” region that likely corresponds to the nucleolus (Figure 3B). We also observed that Rok1-GFP accumulates inside the nucleus in oocytes, excluding somatic follicles and nurse cells (Figure 3B). We subsequently investigated the effect of Rok1 and Rrp5 deletions on ribosomal RNA in multicellular eukaryotes. As shown in Figure 3C, qPCR analysis of the mature rRNAs showed that depletion of *rok1* and *rrp5* significantly decreased the level of 18S, 28S, and 5.8S rRNA in *rok1^141/141^*, *rok1^167/167^*, and *rrp5^4-2/4-2^* mutants (Figure 3C). In addition, using anti-sense probes for 18S and 28S rRNAs via in-situ hybridization (FISH) experiments, we observed that the probes showed weaker fluorescence in the nucleus, and stronger signals in the cytoplasm (Figure 3D). As expected, *rok1^167/167^* showed weaker fluorescence for 18S and 28S rRNA compared with *rok1^167^/TM6B* in third-instar larvae (Figure 3D–F).

### 2.3. Loss of Rok1 Leads to Stalled Ribosome Assembly and Pre-rRNA Processing in the Nucleolus

**Figure 4:**
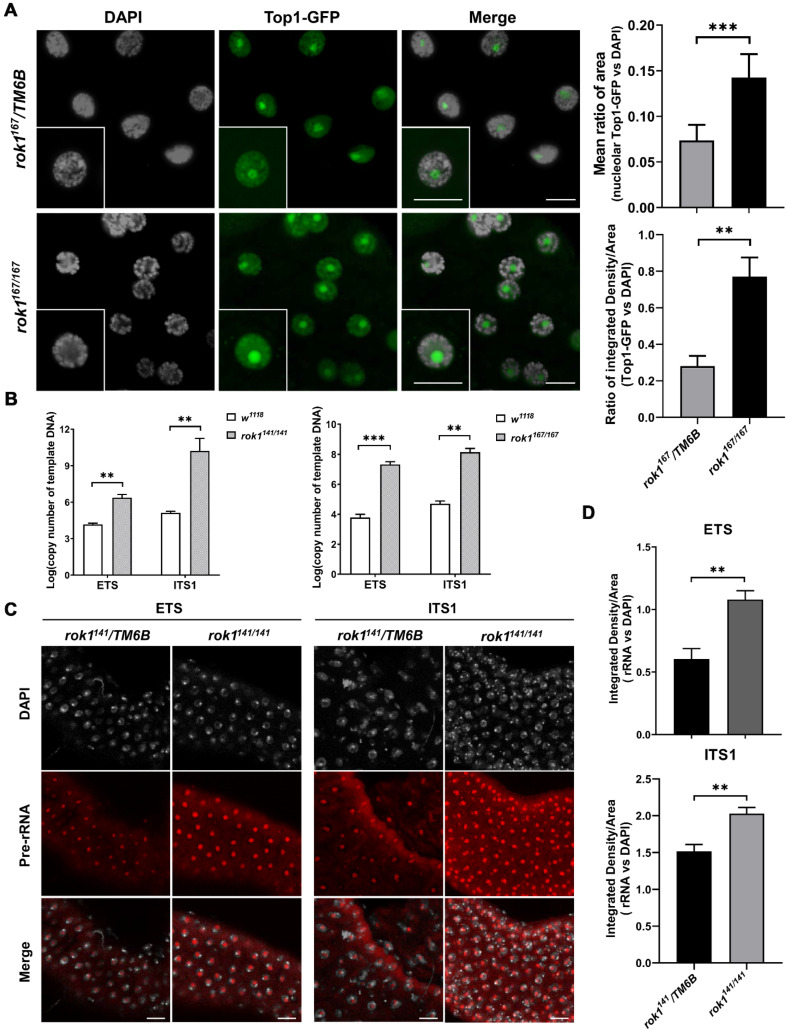
Loss of Rok1 leads to stalled ribosome assembly and pre-rRNA processing in the nucleolus. (**A**) Localization of Top1 in the salivary gland of *rok1^167/167^* mutants. Salivary glands from third-instar larvae of *rok1^167^/TM6B* and *rok1^167/167^* were dissected. Scale bar represents 40 μm. Amplified areas marked with solid rectangles are shown as inserts. Mean ratio of area from salivary gland cells in each genotype is provided in panels to the right. Each point represents the mean ± SD (*n* = 38). *** indicate significant differences between the two groups at *p* < 0.001 (unpaired *t*-test, SPSS). (**B**) The expression levels of ETS and ITS1 in *rok1* mutants. Thirty second-instar larvae of *w^1118^, rok1^141/141^*, and *rok1^167/16^^7^* were pooled as one sample. The initial copy numbers of ETS and ITS1 were calculated from the CT value of each cDNA sample after adjusting by the copy number of *Act5C*. Each point represents the mean ± SD (*n* = 3). ** and *** indicate significant differences between the two groups at *p* < 0.01 and *p* < 0.001 levels, respectively (unpaired *t*-test, SPSS). (**C**) FISH analyses of pre-rRNA localization using ETS and ITS1 probes in salivary glands. Salivary glands were dissected from second-instar larvae of *rok1^141^/TM6B* and *rok1^141/141^*. Strong signals in the nucleolus were shown, with DNA in white and pre-rRNA in red. Scale bar indicates 40 μm. (**D**) Mean ratios of fluorescence density of ETS and ITS1. Each point represents the mean ± SD (*n* = 9). ** and *** indicate significant differences between the two groups at *p* < 0.01 and *p* < 0.001 levels (unpaired *t*-test, SPSS).

Rok1′s function in pre-rRNA processing has been extensively studied in yeast [21,22]. Its function in development is less clear. We speculated that abnormal pre-rRNA processing is a key reason underlying nucleolar enlargement. First, we observed that stronger Top1-GFP fluorescence was produced in *rok1^167/167^* mutants, displaying a larger fluorescent area and higher intensity (Figure 4A). DNA topoisomerase I (Top1) has been proven to mark rDNA loci in larval cells and is responsible for rDNA transcription [23]. Our results suggest that the loss of Rok1 might induce excessive pre-rRNAs in the nucleolus. To further study whether the excessive pre-rRNAs are caused by the accumulation of pre-rRNA intermediates, we determined the relative levels of transcripts containing the external transcribed spacer (ETS) and the first internal transcribed spacer (ITS1) regions. ETS, the first region to be processed, is usually representative of the pre-rRNA transcription rate [15]. The levels of ETS-containing intermediate precursors were significantly increased in Rok1 mutants, suggesting excessive transcription of pre-rRNA in larval cells (Figure 4B). When ITS1 levels were normalized to ETS levels, they also showed approximately 100% and 50% increases in ITS1-containing intermediate precursors in *rok1^141/141^* and *rok1^167/167^*, respectively. FISH experiments in salivary glands showed ETS- and ITS1-containing pre-rRNAs produced in the nucleolus, and the fluorescence of these pre-rRNAs was significantly increased in the nucleolus of the *rok1^141/141^* mutant (Figure 4C,D). All these results indicate that there is a stalled ribosome assembly and pre-rRNA processing in the nucleolus.

### 2.4. Loss of Rok1 Inhibits Mitosis in the Brain

**Figure 5:**
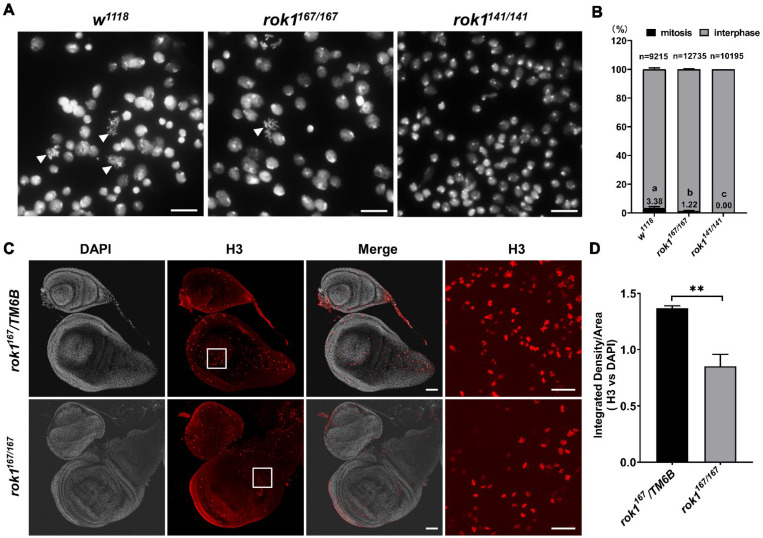
Loss of Rok1 inhibits mitosis in the brain. (**A**) Squashed brains from *rok1* mutant larvae. Brains from third-instar larvae of *w^1118^* and *rok1^167/167^*, and second-instar larvae of *rok1^141/141^* were dissected. Chromosomes marked with an arrow indicate mitosis. Scale bar indicates 10 μm. (**B**) Quantification of the “mitosis” phenotype in each genotype. Different letters indicate significant differences in percentages of cells in mitosis between various groups (*p* < 0.05, LSR, SPSS). (**C**) Immunostaining of wing discs with an anti-phospho-Histone H3 antibody in *rok1* mutant. Wing discs of *rok1^167^/TM6B* and *rok1^167/167^* from third-instar larvae were dissected. Images of the areas marked with solid rectangles are shown as inserts on the right. Scale bar indicates 20 μm. (**D**) Mean ratios of fluorescence density of H3. Each point represents the mean ± SD (*n* = 9). ** indicate significant differences between the two groups at the *p* < 0.01 (unpaired *t*-test, SPSS).

Rok1 has been reported to affect the regulation of the cell cycle in yeast cells [24]. To assess the role of Rok1 in cell division in multicellular eukaryotes, we observed mitosis in the brains of rok1 mutants. By analyzing mitotic chromosomes from the nuclei of *rok1* mutations, we discovered that 1.22% of *rok1^167/167^* chromosomes were in mitosis (*n* = 12,735) compared with 3.38% in the wildtype (*n* = 9215). Mitosis was not seen in *rok1^141/41^* chromosomes (*n* = 10,195) (Figure 5A,B). Considering the operation error due to chromosome squashing, a commercial anti-phospho-Histone H3 antibody was used to immunostain wing discs in rok1 mutants. Generally, Histone H3 phosphorylation is used as a marker of mitosis [25]. As shown in Figure 5C, H3 fluorescence is significantly reduced in the *rok1^167/167^* mutant compared with that of the wildtype (Figure 5C,D). Since second-instar larvae are too small for wing disc dissection, the central nervous system (CNS) was observed. No H3 signal was found (data not shown). These results suggest that the depletion of Rok1 inhibits mitosis in multicellular eukaryotes.

### 2.5. Loss of Rok1 Changes the Cellular Localization of Rrp5 in the Nucleolus

**Figure 6:**
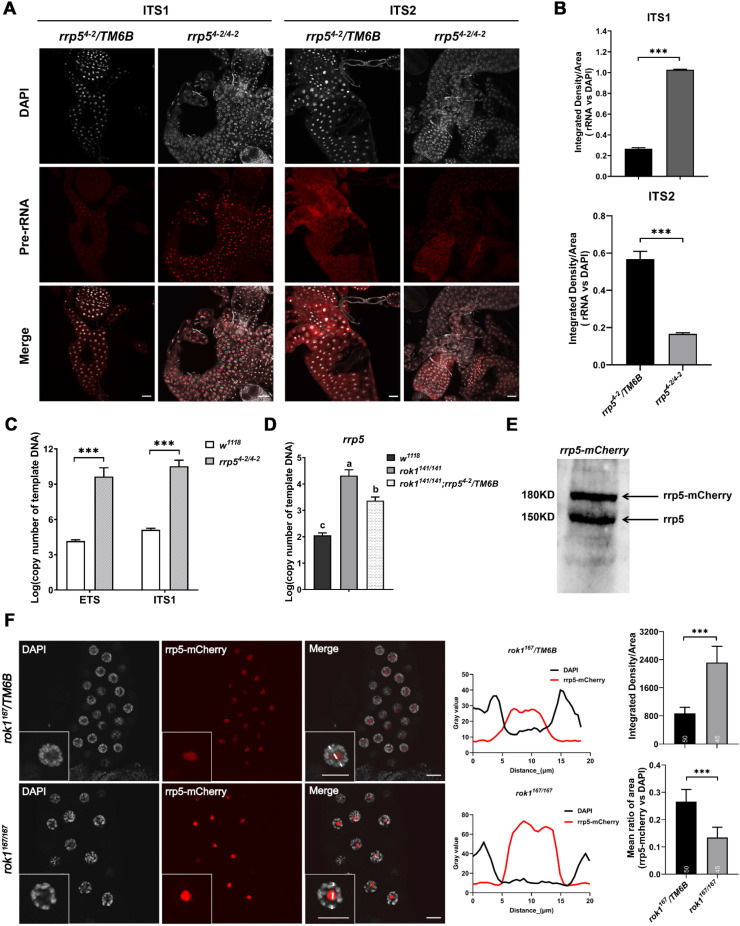
Loss of Rok1 changes the cellular localization of Rrp5 in the nucleolus. (**A**) FISH analyses of pre-rRNA localization using ITS1 and ITS2 probes in malpighian tubules. Malpighian tubules were dissected from second-instar larvae of *rrp5^4-2^/TM6B* and *rrp5^4-2/4-2^*. Strong signals are shown in the nucleolus, with DNA in white and pre-rRNA in red. Scale bar indicates 40 μm. (**B**) Mean ratios of fluorescence density of ITS1 and ITS2. Each point represents the mean ± SD (*n* = 9). *** indicate significant differences between the two groups at the *p* < 0.001 (unpaired *t*-test, SPSS). (**C**) The expression levels of ETS and ITS1 in *rrp5^4-2/4-2^*. Thirty second-instar larvae of *w^111^^8^* and *rrp5^4-2/4-2^* were pooled as one sample. The initial copy numbers of ETS and ITS1 were calculated from the CT value of each cDNA sample after adjusting by the copy number of *Act5C*. Each point represents the mean ± SD (*n* = 3). *** indicate significant differences between the two groups at the *p* < 0.001 (unpaired *t*-test, SPSS). (**D**) The expression levels of *rrp5* in different mutations. Thirty second-instar larvae of *w^1118^, rok1^141/141^*, and *rok1^141/141^; rrp5^4-2^/TM6B* were pooled as one sample. The initial copy numbers of *rrp5* were calculated from the CT value of each cDNA sample after adjusting by the copy number of *Act5C*. Each point represents the mean ± SD from (*n* = 3). (**E**) Rrp5 and Rrp5-mCherry levels were assayed by Western blotting. Total protein extracts (10 ug) from the embryos of *w^1118^* and *rrp5-mCherry* were loaded onto 8% SDS-PAGE gels. Arrows indicate the corresponding bands. (**F**) Localization of Rrp5 in salivary glands of *rok1^167/167^* mutants. Salivary glands from third-instar larvae of *rok1^167^/TM6B* and *rok1^167/167^* were dissected. Scale bar represents 40 μm. Amplified areas marked with solid rectangles are shown as inserts. Thirty-five cells for each genotype were analyzed and summarized in the middle, using a method described in Billmyre et al. [27]. Mean ratio of area and fluorescence density of Rrp5-mCherry in each genotype are provided to the right. Each point represents the mean ± SD (*n* = 35, for area; *n* = 9, for density). *** indicate significant differences between the two groups at the *p* < 0.001 level (unpaired *t*-test, SPSS).

In yeast, Rrp5 binds to pre-40S subunit precursors (SSU), then subsequently binds to the pre-60S subunit precursors (LSU) after release from SSU [21]. We, therefore, speculated that pre-rRNA intermediates were also disrupted in the *rrp5* mutant. In our first assay, two anti-sense probes of ITS1 and ITS2 for 18S and 28S precursors, respectively, were used in FISH. As expected, *rrp5^4-2/4-2^* showed a significant increase in ITS1 fluorescence, and reduced ITS2 fluorescence compared with *rrp5^4-2^/TM6B* in second-instar larvae (Figure 6A,B). In the second assay (Figure 6C), qPCR analysis of ETS and ITS1 showed that deletion of *rrp5* significantly increased levels of 18S precursors in the *rrp5^4-2/4-2^* mutant (Figure 6C). Interestingly, we found that eliminating a copy of *rrp5* in the *rok1^141/141^* mutant could delay the time to lethality in larvae until the late third-instar stage (Table 1), which might be caused by the partial rescue of the increased *rrp5* level in the *rok1^141/141^* mutant (Figure 6D). To further clarify the possible mechanism shown in Figure 6D, the nuclear localization of Rrp5 in the *rok1* mutant was studied to assess Rok1 and Rrp5 interaction in multicellular eukaryotes. An mCherry-tagged fusion protein was constructed and detected by Western blotting. Bands were observed for Rrp5 and Rrp5-mCherry at the exact predicted sizes (Figure 6E). An increased signal of rrp5-mCherry was consistently observed in the rok1^167/167^ mutant, as expected and in accordance with results in Figure 5D (Figure 6F). Tartakoff et al. found that AFs can relocate between the inner (SSU AF) and outer (LSU AF) layers caused by the inhibition of rRNA processing [26]. We found that in the wildtype, Rrp5 fluorescence was distributed within the nucleolus, indicating co-localization with Rok1. Meanwhile, it was particularly enriched in the core of the nucleolus in the *rok1^167/167^* mutant, where SSU assembly factors are distributed [26] (Figure 6F), suggesting that tee loss of Rok1 changes the cellular localization of Rrp5 in the nucleolus.

## 3. Discussion

Rok1, a putative ATP-dependent RNA helicase of the DEAD-box protein family, is required for 18S rRNA synthesis in yeast [28]. Venema et al. found that the GAL:rok1 strain grew slowly after transfer to a glucose medium, when an essential cellular component was depleted gradually [22]. In our study, *rok1* null mutant *Drosophila* larvae died during the second-instar stage (Table 1), which is similar to that observed in yeast. When considering the complex development found in a multicellular eukaryote, the function of Rok1 was deeply exerted in different tissues and stages. We found that the nucleolus of the *rok1* mutant was significantly enlarged in *Drosophila* salivary glands (Figure 2A), and *rok1* depletion led to stalled ribosome assembly and pre-rRNA processing in the nucleolus (Figure 4). This effect has not been reported in single-cell organisms that lack the developmental decision-making requirements. RNA polymerase I-mediated rDNA transcription driven by dMyc overexpression also causes nucleolar morphological enlargement due to pre-rRNA overexpression, as has been observed in *rok1* and *rrp5* mutants [29]. In mammals, Top1 interacts with RNA polymerase I [30,31]. Top1 is specifically inhibited by camptothecin in HeLa cells, resulting in the inhibition of rRNA synthesis [32]. Studies on yeast have found that Top1 depletion leads to the accumulation of many negative supercoils in rDNAs [33]. As shown in Figure 3A, we also observed strong enrichment of Top1 in nucleoli in *rok1^167/167^* (Figure 4A), suggesting that Top1 is also involved in the pre-rRNA accumulation caused by Rok1 depletion.

Based on previously reported data, a model Rok1 functional cycle during ribosome maturation has been proposed [21]. Ribosome biogenesis is a fundamental cellular process, linked to growth and cell division. Mutation of Rok1 resulted in significant delays in mitotic bud emergence derived from the inhibition of Rok1 expression throughout the cell cycle [24], in accordance with our results seen in *Drosophila* (Figure 5). The depletion of Rok1 led to abnormal processing of 35S pre-rRNA within ITS1 to produce an abnormal 23S pre-rRNA instead of the mature 18S rRNA, which is subsequently degraded [22]. In our study, qPCR and FISH analysis of the mature rRNAs showed that *rok1* depletion significantly decreases levels of 18S, 28S, and 5.8S rRNA in *rok1^141/141^* and *rok1^167/167^* mutants (Figure 3C,D). We speculated that some aspects of rRNA processing differ between higher eukaryotes and their yeast counterparts, although the fundamental ribosome biogenesis pathway is conserved.

Previous studies in yeast have shown that Rrp5 is a key assembly factor in rRNA processing [11]. It simultaneously participates in the processing of small subunit and large subunit precursors and bears primary responsibility for the recruitment of other assembly factors [12], which are required for the transfer of Rrp5 from SSU to LSU by the Rok1 protein. Although Rok1 and Rrp5 proteins are conserved, whether their mechanisms during rRNA processing are conserved among eukaryotes (especially multicellular eukaryotes) has not been confirmed. We obtained homozygote mutants of *rok1* and *rrp5* using the Crispr/Cas9 system (Figure 1A). These mutants showed different degrees of developmental defects. Development in larvae carrying the frameshift mutations *rok1^141/141^* and *rrp5^4-2/4-2^* stagnated during the second-instar stage, and development in the *rok1^167/167^* mutant stagnated in the third-instar stage (Table 1). Compared with the wildtype, pre-rRNA levels increased significantly in the *rok1* and *rrp5* mutants, while levels of mature rRNAs including 18S, 28S, and 5.8S rRNAs decreased significantly (Figure 3, Figure 4, and Figure 6), indicating that rRNA processing can be blocked by individual loss of Rok1 or Rrp5. In yeast, ATP-bound Rok1 stabilizes Rrp5 binding to 40S ribosomes and is required for the transfer of Rrp5 from SSU to LSU [21]. In our study, *rrp5^4-2/4-2^* showed a significantly increased fluorescence signal in ITS1, and reduced ITS2 fluorescence by FISH compared with *rrp5^4-2^/TM6B* in second-instar larvae (Figure 6A,B), and qPCR analysis of ETS and ITS1 levels showed that *rrp5* depletion significantly increased the level of 18S precursors in the *rrp5^4-2/4-2^* mutant (Figure 6C). These results suggest that the ITS1 accumulation is caused by Rrp5 depletion. Although pre-rRNA biogenesis stalls in the nucleolus, the loss of Rrp5 subsequently blocks LSU processing, resulting in a significantly reduced ITS2 signal (Figure 6A). Moreover, we observed an obvious shift in Rrp5 localization in *rok1* mutants (Figure 6F). Tartakoff et al. found that nucleolar assembly factors (AFs) associated with rRNAs localize to the coaxial layers of the nucleoli in yeast. AFs can relocate between the inner (SSU AF) and outer (LSU AF) layers caused by the inhibition of rRNA processing [26]. Rrp5 has been reported both in the inner and outer layers of nucleoli for SSU and LSU maturation. In *rok1^167/167^* mutants, Rrp5 was localized and highly enriched in the inner layer of the nucleolus, in which SSU assembly factors are distributed (Figure 6F). We thus assume that the interaction between Rok1 and Rrp5 in eukaryotes is highly conserved.

**Figure 7:**
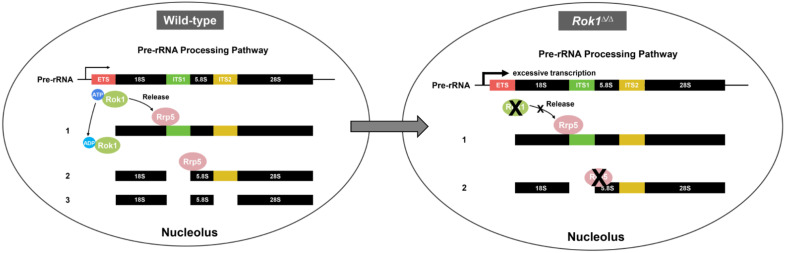
Model of Rok1 functions on ribosomal rRNA processing in *Drosophila*.

In our study, we used a combination of genetics and developmental experiments to show that Rok1 and Rrp5, which are localized at the nucleolus, play key functions in ribosome assembly in *Drosophila* (Figure 7).

## 4. Materials and Methods

### 4.1. Drosophila Stocks

Rok1 and Rrp5 mutations were generated as described in Section 4.2. *Drosophila* stocks were raised on a normal medium containing glucose, corn, sucrose, yeast, and agar under standard laboratory conditions. Stocks including *w^1118^*, nos-Cas9, and Sb/TM6B, were obtained from the Bloomington *Drosophila* stock center. The Fib-GFP and Top1-GFP stocks were obtained as previously described [23]. The rok1-GFP and rrp5-mCherry stocks were generated as described in Section 4.3.

### 4.2. Crispr/Cas9-Mediated Mutations of Rok1 and Rrp5

Two mutant alleles of *rok1* (CG5589) (*rok1^141/141^* and *rok1^167/167^*) and *rrp5* (CG5728) (*rrp5^4-2/4-2^* and *rrp5^11-/11-1^*) were generated in a *w^1118^* background using the CRISPR-Cas9 system. The Cas9 protein and gRNA derived by vasa and U6 promoters were expressed by transgenes inserted into the *Drosophila* genome, respectively [34]. The target gRNA was designed by the online page: http://tools.flycrispr.molbio.wisc.edu/targetFinder/. The target sequences 5′-CCTGTGCACCCACTGGATC (for *rok1*) and 5′- AGCTCTTCCAGTTGGGCCG (for *rrp5*) were chosen and cloned into a pUAST-attB plasmid for phiC31-mediated germline transformation to generate offspring with red eyes (G0). Cross schemes and mutation selections were performed as described in Supplementary Figure S1. PCR primers used for mutation detection are listed in Supplementary Table S1.

### 4.3. Construction of rok1-GFP and rrp5-mCherry Transgenic Lines

To construct transgenic lines carrying *rok1-GFP* and *rrp5-mCherry*, DNA fragments of the whole genomic region containing *rok1* and *rrp5* were cloned into the pUAST-attB vector by gap repairs based on “Seamless ligation cloning”, and the GFP and mCherry genes were subsequently inserted at the 3′ ends of the Rok1 and Rrp5 coding regions, respectively, upstream of the stop codon by recombineering [35]. Rok1-GFP and rrp5-mCherry fly lines were generated by PhiC31 integrase-mediated germline insertions on chromosome III (86F) and chromosome II (25C), respectively. Because the X chromosome of 86F and 25C strains contains ΦC31, which induces green fluorescence, lines were hybridized with *w^1118^* to remove this background. PCR primers are listed in Supplementary Table S1.

### 4.4. Quantitative PCR

Total RNA from thirty second-instar larvae of *w^1118^, rok1^141/141^*, *rok1^167/167^*, *rrp5^4-2/4-2^*, *rok1^141/141^,* and *rrp5^4-2^/TM6B* was extracted using TRIzol reagent (Invitrogen, Carlsbad, California, USA). cDNA was generated from 1 µg of total RNA using a PrimeScript^TM^ RT reagent kit (TaKaRa, Shiga, Japan) for the quantitation of mRNAs including *rok1*, *rrp5*, 18S rRNA, 28S rRNA, 5.8S rRNA, ETS, and ITS1 by quantitative real-time PCR. A Bio-Rad CFX96 Real-Time System (Bio-Rad, Hercules, CA, USA) and a KAPA SYBR FAST Universal qPCR Kit (Kapa Biosystems, Boston, MA, USA) were used for quantitative real-time PCR (qPCR). The cDNA standard for each gene was diluted to different concentrations and used as templates for PCR reactions. *Act5C* (accession number CG4027) was used as an internal control gene. The standard curves of *Act5C*, *rok1*, *rrp5*, 18S rRNA, 28S rRNA, 5.8S rRNA, ETS, and ITS1 were drawn with the logarithm of the copy number of template DNA as the abscissa and the measured CT value as the ordinate. The initial copy number of *rok1*, *rrp5*, 18S rRNA, 28S rRNA, 5.8S rRNA, ETS, and ITS1 was calculated from the CT value of each cDNA sample after adjustment by the copy number of *Act5C*. Each cDNA was displayed for three biological replicates. *P*-values were indicated using Student’s *t*-test. The primers are listed in Supplementary Table S1.

### 4.5. Live Fluorescent Imaging

For rok1-GFP live imaging, salivary glands from third-instar larvae of *w^1118^*, ovaries, and testes from adult flies were dissected in cold PBS. For Fib-GFP live imaging, salivary glands from second-instar larvae of *rok1^141/141^* and *rrp5^4-2/4-2^* and third-instar larvae of *rok1^167^/TM6B* and *rok1^167/167^* were dissected, respectively. For Top1-GFP live imaging, salivary glands from third-instar larvae of *rok1^167^/TM6B* and *rok1^167/167^* were dissected. For rrp5-mCherry live imaging, salivary glands from third-instar larvae of *rok1^167^/TM6B* and *rok1^167/167^* were dissected. Thirty biological replicates were detected for each mutation. The tissues were imaged by a Zeiss Axio Image A2 microscope.

### 4.6. Fluorescence In Situ Hybridization (FISH)

Salivary glands were dissected in PBS from second-instar larvae of *rok1^141^/TM6B* and *rok1^141/141^*, and third-instar larvae of *rok1^167^/TM6B* and *rok1^167/167^*. Malpighian tubules were dissected from second-instar larvae of *rrp5^4-2^/TM6B* and *rrp5^4-2/4-2^* in PBS. Tissues were fixed in 4% paraformaldehyde for 4 h. After washing three times with 2 × SSCT (0.1% Triton X-100), they were rehydrated using an ethanol gradient. After air drying, pre-hybridization was performed in 25% formamide, 5 × Denhards, 5% dextran sulfate, 2 × SSC, 0.2% BSA, and 125 μg tRNA for 2 h at 37 °C. Tissues were incubated at 37 °C overnight with a final probe concentration of 0.5 ng/mL. Post-hybridization washes were performed three times in 0.2 × SSCT for 15 min each. Tissues were then stained with DAPI (0.2 μg/mL in 4 × SSCT) for five min, washed briefly in 4 × SSCT, and allowed to air dry. Fluorescent images were photographed by an Olympus IX83 confocal microscope. Probes are listed in Supplementary Table S1.

### 4.7. Antibodies

Mouse anti-rok1 (CG5589) and anti-rrp5 (CG5728) sera were raised against 333 amino acid fragments of Rok1 (a.a. 121-453) and Rrp5 (a.a. 151-483) as antigens purified from *E. coli*. The Rok1 and Rrp5 antibodies were incubated at 1:1000 and 1:2000 on Western blots, respectively. Embryos were collected and homogenized in RIPA buffer, and an anti-mouse secondary antibody (1:5000 dilution, Abcam) was used.

### 4.8. Immunostaining

Ten wing discs of *rok1^167^/TM6B* and *rok1^167/167^* from third-instar larvae were dissected in the cold PBS buffer. Then, tissues were fixed with a mixture of 3.7% formaldehyde diluted in PBS for 15 min. Heptane was subsequently added for 30 min at room temperature. After washing three times with 1 X PBST (0.1% Triton X-100), a rabbit anti-phospho-Histone H3 (1:200, Sigma, Saint Louis, USA) antibody was incubated overnight at 4 °C. After washing three times with 1 X PBST (0.1% Triton X-100), Alexa Fluor 555 conjugated goat antirabbit IgG (1:200, Abcam) and DAPI were incubated for 1 h. Fluorescent images were photographed by an Olympus IX83 confocal microscope.

### 4.9. Mitotic Chromosome Squash

Brains from second- and third-instar larvae of *w^1118^*, *rok1^167/167^*, and *rok1^141/141^* were dissected in cold PBS buffer following a standard protocol without colchicine treatment [36]. Ten brains from each group were dissected and squashed. Chromosomes were analyzed with a Zeiss Axio Image A2 microscope.

## Figures and Tables

**Table 1:** The lethal phenotypes of *rok1* and *rrp5* mutations.

Genotype	Lethal Stage	Genotype	Lethal Stage
*rok1^141/141^*	2nd instar	*rok1^167/167^; rrp5^4−2/4-2^*	2nd instar
*rok1^167/167^*	pupa	*rok1^141/141^; rrp5^4-2^/TM6B*	Late 3rd instar
*rrp5^4-2/4-2^*	2nd instar	*rok1^141/141^; rrp5^11-1^/TM6B*	2nd instar
*rrp5^11-1/11-1^*	----	*rok1^141^/TM6B; rrp5^4-2/4-2^*	2nd instar
*rok1^141/141^; rrp5^11-1/11-1^*	2nd instar	*rok1^141^/TM6B; rrp5^11-1/11-1^*	----
*rok1^141/141^; rrp5^4-2/4-2^*	2nd instar	*rok1^167/167^; rrp5^4-2^/TM6B*	pupa
*rok1^167/167^; rrp5^11-1/11-1^*	pupa	*rok1^167^/TM6B; rrp5^4-2/4-2^*	2nd instar

This table summarizes the phenotypes of *rok1*, *rrp5* and double mutations. “----“ alive.

## Data Availability

Not applicable.

## Supplementary Materials

The following supporting information can be downloaded at: https://www.mdpi.com/article/10.3390/ijms23105685/s1.
