# Supplementary material for: The DEAD-Box Protein Rok1 Coordinates Ribosomal RNA Processing in Association with Rrp5 in Drosophila
Source: Int J Mol Sci. 2022 May 19;23(10):5685. doi: 10.3390/ijms23105685 (PMC9146779; doi:10.3390/ijms23105685)
Supplement: Supplementary file 1 [file ijms-23-05685-s001.zip › supplemental.pdf]

Cross schemes and mutation selections of *rok1* and *rrp5* mutations

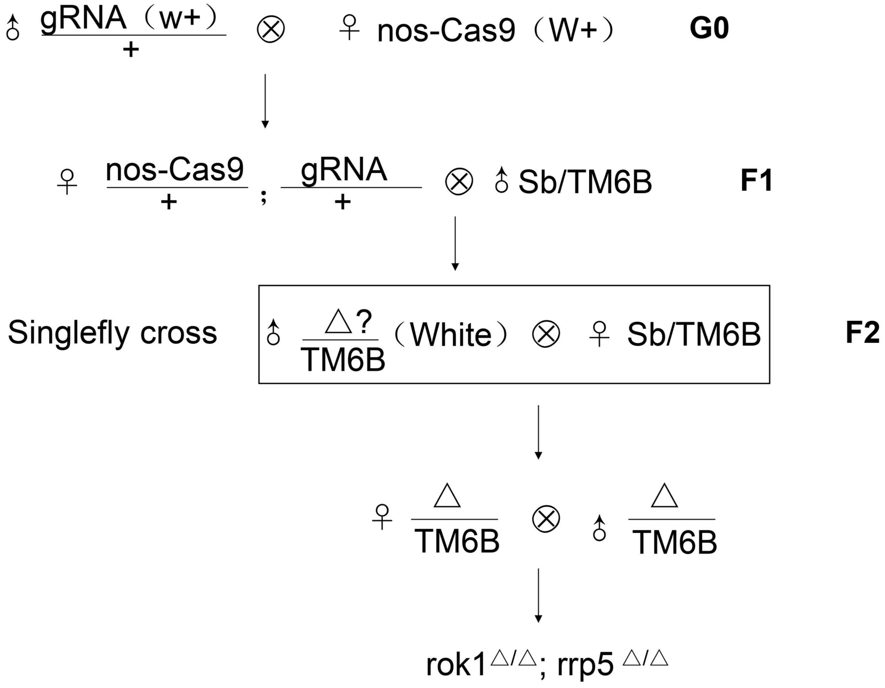

Table S1 PCR primers used in this study

Primers	Primer sequence
For RT-PCR detection	
Qrok1-F	5'-TGCCGAAAGGAGTCAACATC-3'
Qrok1-R	5'-TGGGCGGAAAGTCGTAGTT-3'
Qrrp5-F	5'-TGATGCGACCACCAATGACT-3'
Qrrp5-R	5'-AACTGCCTCGTGCTTGCTAC-3'
Q18S-F	5'-GGCTAAAACCAAGCGATCGC-3'
Q18S-R	5'-CTCCCTCTCCGGAATCGAAC-3'
Q28S-F	5'-GTCAGGGGAAACCCTGATGG-3'
Q28S-R	5'-ATCGTTTCGACCCTAAGGCC-3'
Q5.8S-F	5'-CTAGGCGGTGGATCACTCGG-3'
Q5.8S-R	5'-CAGCATGGACTGCGATATGC-3'
QETS-F	5'-GCTCCGCGGATAATAGGAAT-3'
QETS-R	5'-ATATTTGCCTGCCACCAAAA-3'
QITS1-F	5'-TTATTGAAGGAATTGATATATGCC-3'
QITS1-R	5'-ATGAGCCGAGTGATCCAC-3'
QAct5C-F	5'- GCGTGGTATCCTCACCCTGA-3'
QAct5C-R	5'- GGTTGGCCTTGGGGTTCAGC-3'
For mutant detection	
rok1-F	5'-GACACTATCCCCCAAGGAAT-3'
rok1-R	5'-TCCGCACCATGTTTCTGCTT-3'
rrp5-F	5'-AGCTACGAAACCGAGGAACA-3'
rrp5-R	5'-TTGTACTCCCGACTCGATGA-3'
For FISH detection	
ETS	5'-ATTCCTATTATCCGCGGAGC-3'
ITS1	5'-GGCATATATCAATTCCTTCAATAA-3'
ITS2	5'-ACCCTCAACCATATGTAGTCCAAGCAGCAC-3'
18S rRNA	5'-CTGTCGTCGGTACAAGACCA-3'
28S rRNA	5'-CATCAGGTGATCGAAGATCCTCC-3'
For transgenic constructs	
rok1-GFP-F	5’-AAAGGAAAGGAAATGTAGAGAAAAAAACAAAA AATAAAAATAATCAAACTGGCGGAGGGCGCGCCC TGTGGAACACCTACATCT-3’
rok1-GFP-R	5’-AAACGCAAAACAAGATATTTTTATACACTTTACTTT AATTTCGCGTATCACTTGTACAGCTCGTCCATGC-3’
rrp5-mCherry-F	5’-AAATTTTGTTATTTAAAACGCTAAATTACACA GCTCCCGGATGGTGAGCAAGGGCGAGGA-3’

rrp5-mCherry-R	5’-GGATTGTCCCGCCTCGGGGAAAGCTTTTCTC GTTCGGCACGGCGCGCCAGCCAGTATACAC-3’

F: Forward; R: Reverse
